# Calcium tunneling through the ER and transfer to other organelles for optimal signaling in *Toxoplasma gondii*

**DOI:** 10.1101/2024.08.15.608087

**Published:** 2024-08-15

**Authors:** Zhu-Hong Li, Beejan Asady, Le Chang, Miryam Andrea Hortua Triana, Catherine Li, Isabelle Coppens, Silvia N.J. Moreno

**Affiliations:** 1 Center for Tropical and Emerging Global Diseases, Department of Computes Science, University of Georgia, Athens, Georgia 30602; 2 Department of Molecular Microbiology and Immunology, Johns Hopkins Bloomberg School of Public Heath, Baltimore, MD 21205; 3 Department of Cellular Biology, University of Georgia, Athens, Georgia 30602

**Keywords:** Calcium, endoplasmic reticulum, membrane contact sites, Toxoplasma gondii, SERCA-Ca^2+^-ATPase, calcium tunneling

## Abstract

Ca^2+^ signaling in cells begins with the opening of Ca^2+^ channels in either the plasma membrane (PM) or the endoplasmic reticulum (ER) and results in a dramatic increase in the physiologically low (<100 nM) cytosolic Ca^2+^ level. The temporal and spatial Ca^2+^ levels are well regulated to enable precise and specific activation of critical biological processes. Ca^2+^ signaling regulates pathogenic features of apicomplexan parasites like *Toxoplasma gondii* which infects approximately one-third of the world’s population. *T. gondii* relies on Ca^2+^ signals to stimulate traits of its infection cycle and several Ca^2+^ signaling elements play essential roles in its parasitic cycle. Active egress, an essential step for the infection cycle of *T. gondii* is preceded by a large increase in cytosolic Ca^2+^ most likely by release from intracellular stores. Intracellular parasites take up Ca^2+^ from the host cell during host Ca^2+^ signaling events to replenish intracellular stores. In this work, we investigated the mechanism by which intracellular stores are replenished with Ca^2+^ and demonstrated a central role for the SERCA-Ca^2+^-ATPase to keep not only the ER filled with Ca^2+^ but also acidic stores. We also show mitochondrial Ca^2+^ uptake, by transfer of Ca^2+^ from the ER most likely through membrane contact sites. We propose a central role for the ER in tunneling of calcium from the extracellular milieu through the ER to other organelles.

## INTRODUCTION

*Toxoplasma gondii* is an intracellular parasite from the Apicomplexan Phylum that infects approximately one third of the world population (Weiss and Dubey, 2009). During the initial infection with *T. gondii*, the parasite engages in multiple rounds of a lytic cycle, which consists of invasion of host cells, replication inside a parasitophorous vacuole (PV), exit from the host cell causing its lysis followed by invasion of a new host cell (Black and Boothroyd, 2000; Blader et al., 2015). Cytosolic Ca^2+^ ([Ca^2+^]_c_) fluctuations precede the activation of several key steps of the *T. gondii* lytic cycle like motility, attachment, invasion, and egress ([Ca^2+^]_c_) (Lourido and Moreno, 2015; Hortua Triana et al., 2018). Egress from the host cell is an essential step for the infection cycle of *T. gondii* (Bisio and Soldati-Favre, 2019) and it was shown that it is preceded by a cytosolic Ca^2+^ increase (Endo et al., 1982; Borges-Pereira et al., 2015). Extracellular Ca^2+^ entry was demonstrated in extracellular (Pace et al., 2014; Hortua Triana et al., 2024) and intracellular replicating tachyzoites (Vella et al., 2021). This activity was highly regulated and recent work from our lab showed that a TRP-like activity was involved (Marquez-Nogueras et al., 2021).

Ca^2+^ signaling is part of the signaling pathways that regulate a large number of cellular functions (Clapham, 2007). All cells express a variety of channels, transporters, and Ca^2+^ pumps, located at the plasma membrane (PM) and/or intracellular organelles (endoplasmic reticulum (ER), acidic stores, and mitochondria) that regulate/control the concentration of cytosolic Ca^2+^ ([Ca^2+^]_c_). A high cytosolic Ca^2+^ concentration for sustained lengths of time, is toxic to cells and may result in their death (Bootman and Bultynck, 2020).

In *T. gondii*, both Ca^2+^ entry through the plasma membrane and influx from intracellular stores like the ER may initiate a cascade of signaling events important for the stimulation of the biological steps of the parasite lytic cycle (Lourido and Moreno, 2015; Hortua Triana et al., 2018). Ca^2+^ oscillations were observed in motile parasites loaded with fluorescent Ca^2+^ indicators, (Lovett and Sibley, 2003) as well as expressing Genetically Encoded Calcium Indicators (GECIs) (Borges-Pereira et al., 2015). The significance of Ca^2+^ signals during all stages of the lytic cycle has been demonstrated, but little is known about the mechanism by which intracellular stores contribute to cytosolic Ca^2+^ signals and downstream regulation.

The ER, an exclusive organellar feature of eukaryotic cells, is the main store for Ca^2+^ in most eukaryotic cells and it is widely accepted that: the ER is functionally heterogeneous with Ca^2+^ binding proteins and Ca^2+^ pumps and channels having a nonuniform distribution resulting in the presence of distinct subdomains within the organelle (Papp et al., 2003); the ER facilitates Ca^2+^ tunneling through its lumen as a mechanism of delivering Ca^2+^ to targeted sites without activating inappropriate processes in the cell cytosol (Petersen et al., 2017); and the ER is ubiquitously distributed and is in close contact with all cellular organelles and the plasma membrane (PM) (Spang, 2018). Over the past decade a new paradigm has emerged that seeks to decipher how subcellular organelles communicate with each other in order to coordinate activities and efficiently distribute ions and lipids within the cell. Numerous observations have highlighted the presence of tight, stable and yet non-fusogenic associations between organellar membranes which have since become known as membrane contact sites (MCSs) (Phillips and Voeltz, 2016).

The secretory pathway of *T. gondii* is organized in a highly polarized manner with the ER being an extension of the nuclear envelope (Hager et al., 1999; Tomavo et al., 2013). The ER at the apical surface of the nuclear envelope continues with the Golgi stacks and then the secretory organelles, micronemes and rhoptries, which are unique to the apicomplexan phylum (Hager et al., 1999). These organelles perform important functions relevant to a successful lytic cycle like host cell attachment, invasion, and establishment of the parasitophorous vacuole (PV). Cytoplasmic Ca^2+^ increases, due to efflux from the ER, have been reported to initiate responses like microneme secretion (Carruthers and Sibley, 1999; Nagamune et al., 2007b), conoid extrusion (Del Carmen et al., 2009), invasion (Vieira and Moreno, 2000; Lovett and Sibley, 2003) and egress (Arrizabalaga and Boothroyd, 2004). These responses require precise spatiotemporal control of diverse targets and suggests the presence of distinct systems to deliver Ca^2+^ to specific locations rather than allowing global increases, which would activate unnecessary and potentially detrimental signaling events (Huet and Moreno, 2023).

In order to concentrate Ca^2+^ ions, the ER utilizes a SERCA-Ca^2+^-ATPase, a transmembrane P-type ATPase, that couples ATP hydrolysis to the transport of ions across biological membranes and against a concentration gradient. SERCA pumps can be inhibited by various inhibitors including the very potent and highly specific thapsigargin (TG) (Thastrup et al., 1990; Sagara and Inesi, 1991).

*T. gondii* expresses a SERCA Ca^2+^-ATPase (TgSERCA), which possess SERCA conserved domains and Ca^2+^ binding sites and ATPase residues (Nagamune et al., 2007a). The function of TgSERCA was determined by rescue experiments of yeast cells defective in Ca^2+^-ATPases and by its specific inhibition by TG (Nagamune et al., 2007a). TgSERCA was mainly localized to the ER of *T. gondii* but also showed a distinct distribution in extracellular parasites, where the protein was partially found in ER vesicles in the apical region near micronemes (Nagamune et al., 2007a). This distribution pattern was different to the one obtained with the transient transfection of GFP-HDEL (an ER marker), which was retained near the nuclear envelope, suggesting an uneven distribution of ER markers in extracellular parasites. The authors suggested that this distribution to the apical end may be important for rapid release and effective recovery of cytosolic Ca^2+^, events that likely govern both motility and microneme secretion (Nagamune et al., 2007a; Nagamune et al., 2007b).

In this work we characterize the mechanism by which the ER efficiently takes up Ca^2+^ promptly after the ion enters the cytosol from the extracellular milieu. We also characterize the role of the ER in transferring Ca^2+^ to other stores. We propose a model in which the ER is the distribution center for Ca^2+^ and due to the high Ca^2+^ affinity of the SERCA Ca^2+^-ATPase the ER is highly efficient at storing Ca^2+^ and transferring it to other compartments.

## RESULTS

### Extracellular Ca^2+^ predominantly fills the endoplasmic reticulum and acidic stores

We first set up experiments to follow the destination of Ca^2+^ taken up by extracellular tachyzoites from the extracellular milieu into their cytosol. Tachyzoites were loaded with the ratiometric Ca^2+^ indicator Fura-2 and incubated in Ringer buffer containing 100 μM EGTA to chelate extracellular Ca^2+^, preventing its uptake, and investigated the release of intracellular Ca^2+^ pools by different inhibitors.

**Figure 1:**
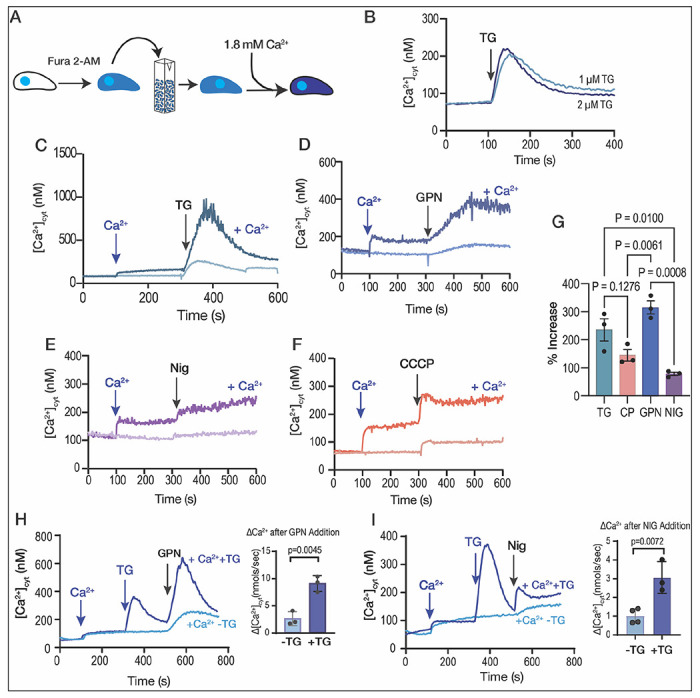
The role of extracellular calcium in the filling of intracellular stores. **A**, scheme depicting the Fura-2-AM loading and the experimental set-up. **B**, *T. gondii* tachyzoites loaded with Fura-2 were in suspension in Ringer buffer with 100 μM EGTA. Thapsigargin (TG) was added at 100 seconds, at two different concentrations (1 and 2 μM). **C**, Similar experimental set-up to the one shown in B. 1.8 mM CaCl_2_ was added to the suspension at 100 sec, followed by 2 μM TG at 300 sec (*dark blue trace*). The light blue trace shows the same experiment without the addition of CaCl_2_. **D**, similar to C but using 40 μM GPN instead of TG. **E**, same experimental set-up to the one shown in C but adding the potassium ionophore nigericin, 10 μM. **F**, same experimental set-up to the one shown in C but using the mitochondrial uncoupler CCCP. **G**, quantification of the % increase of cytoplasmic calcium by adding extracellular calcium prior to each indicated reagent (TG, thapsigargin; CP, CCCP; NIG, nigericin). **H**, cytosolic calcium measurements following the addition of 1.8 mM extracellular calcium, 1 μM TG prior to the addition of GPN, 40 μM. I, cytosolic Calcium measurements following the addition of 1.8 mM extracellular Calcium, 1 μM TG prior to the addition of 10 μM NIG. The quantification shows the comparison of the cytosolic increase with and without the addition of TG. Data are presented as mean ± SD for G, H and I. *p value*: unpaired two tailed t test performed in all comparisons.

When thapsigargin (TG), which inhibits the activity of SERCA, was added, it prevented the re-uptake of Ca^2+^ into the ER and unmasked the leakage of Ca^2+^ from the ER into the cytosol, causing a Ca^2+^ cytosolic increase of approximately 160 nM (Fig. 1B **and S1A**). We compared this with the effect of ionomycin (IO), a Ca^2+^/H^+^ ionophore which acts on neutral intracellular Ca^2+^ stores, inducing depletion of Ca^2+^, mainly from the ER (Smith et al., 1989). Exposure of *T. gondii* to 1 μM ionomycin caused a cytosolic increase of approximately 700-1100 nM Ca^2+^ (**Fig. S1A-B**). We next tested another SERCA inhibitor, cyclopiazonic acid (CPA), with a different mode of action than TG. CPA caused a lower Ca^2+^ cytosolic increase than the one caused by TG, possibly implying less specificity toward TgSERCA **(Fig. S1B)**. Exposure of parasites to TG before CPA ablated CPA’s effect, but this did not occur when adding CPA prior to TG **(Fig. S1C-D)**. Although TG does a better job at depleting the ER of Ca^2+^, the increase of cytosolic Ca^2+^ after adding TG is un-impressive when compared to the effect of IO **(Fig. S1A)**. This result could indicate the presence of other calcium stores not inhibited by TG or simply that TG was not very efficient at blocking TgSERCA.

Next, we tested the effect of Ca^2+^ entry in the filling of intracellular stores by measuring the cytosolic Ca^2+^ increases in response to inhibitors after pre-exposing the parasite suspension to extracellular Ca^2+^ (Fig. 1C–F, *dark traces*). Addition of 1.8 mM Ca^2+^ caused a cytosolic increase due to influx through a partially characterized plasma membrane pathway (Fig. 1C, *dark blue trace*) (Pace et al., 2014). This Ca^2+^ influx appeared to be immediately taken up by the ER as the response to TG was much greater in parasites previously exposed to extracellular Ca^2+^ (compare light and dark blue traces) (Fig. 1C). We next tested the lysosomotropic agent glycyl-L-phenylalanine-naphthylamide (GPN), which mobilizes Ca^2+^ from acidic organelles (Haller et al., 1996; Lloyd-Evans et al., 2008; Miranda et al., 2010; Yuan et al., 2021) and observed a similar pattern (Fig. 1D). The cytosolic Ca^2+^ increase upon addition to GPN in parasites previously exposed to extracellular Ca^2+^ was much greater than in cells not exposed to extracellular Ca^2+^ (Fig. 1D, *compare light and dark blue traces*). A similar phenomenon was observed when adding nigericin (acting on acidic stores) or CCCP (most likely acting on mitochondria) (Figs 1E–F, *dark purple and orange traces, respectively*). These data indicate that the ER, mitochondrion, and PLVAC and other acidic stores, all appear to have the ability to access extracellular Ca^2+^. The ER displayed the highest capacity to access a large portion of extracellular Ca^2+^, with TG inducing close to ~500-900 nM of Ca^2+^ release after pre-exposure to Ca^2+^ and only 210 nM Ca^2+^ without Ca^2+^ pre-exposure (Fig. 1C).

In summary, upon pre-exposure of *T. gondii* to physiological levels of extracellular Ca^2+^, the ER, mitochondria, and acidic stores exhibited the capacity to release many times more Ca^2+^ than without pre-exposure, with the ER and GPN-sensitive stores displaying the greatest response (Fig 1G).

We next hypothesized that if the ER is the most efficient organelle to access extracellular Ca^2+^ and pass it to the other organelles, it should be possible to see an increased response to agonists like GPN and nigericin after inhibiting SERCA with TG permitting the buildup of Ca^2+^ on the cytosolic side of the ER membrane. For the next experiment we added Ca^2+^ to fill the stores, followed by TG to cause Ca^2+^ efflux from the ER, and then GPN (Fig. 1H). We compared the response to GPN between plus and minus the addition of TG and observed that the cytosolic increase caused by GPN when preceded by TG was significantly larger (Fig. 1H). We followed the same approach with nigericin and observed that the response increased after the addition of TG (Fig. 1I). These experiments support our hypothesis that extracellular Ca^2+^ is used to fill the ER and from the ER Ca^2+^ can be distributed to other organelles.

### Ca^2+^ uptake by the SERCA-Ca^2+^ ATPase in permeabilized tachyzoites

The previous results highlighted an important role for the ER in taking up Ca^2+^ from the cytoplasm after an increase due to influx from the extracellular milieu. We believe that this ER uptake, essential for keeping stores filled with Ca^2+^, is due to the high Ca^2+^ affinity of TgSERCA. The SERCA functions as an essential mechanism for the ER to maintain its Ca^2+^ levels, an especially important function considering the leakage of Ca^2+^ from the ER into the cytosol which occurs constitutively and passively (Camello et al., 2002).

**Figure 2:**
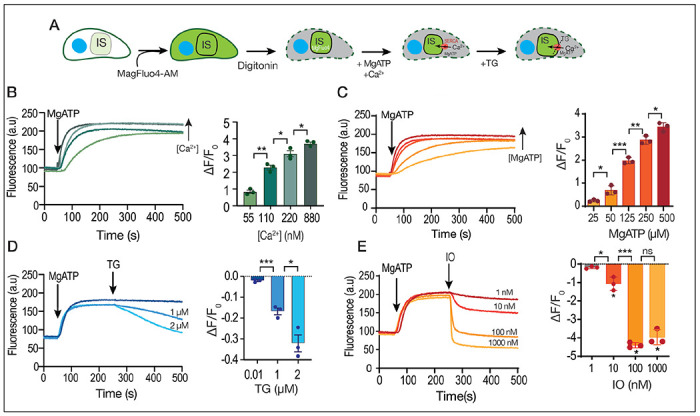
Ca^2+^ uptake by intracellular stores. **A**, Scheme showing the loading with MagFluo4-AM followed by permeabilization with digitonin of a *T. gondii* tachyzoite (RH parental strain) suspension (IS, intracellular store). **B**, Fluorescence measurements (see Materials and Methods for specifics) of the suspension of parasites loaded with MagFluo4. MgATP was added at 500 μM at 50 seconds. The bar graph shows the quantification of the slope of the increase in fluorescence after adding the SERCA substrate MgATP. The concentration of free calcium was varied, and it is indicated. The calculation of free calcium was done with MaxChelator. **C**, similar experimental set-up to the one shown in B but the concentration of MgATP was varied at the concentrations indicated in the bar graph. Quantification of the slope of fluorescence increase after adding MgATP is shown in the bar graph. **D**, this experiment was done with 500 μM MgATP and 220 nM CaCl_2_. Thapsigargin (TG) was added to inhibit SERCA causing calcium to be released from the store. The concentrations used are indicated. The bar graph shows the negative slope after the addition of TG. **E**, similar to D, but adding ionomycin (IO). The concentrations used are indicated and the slopes were measured after the addition of IO. Data are presented as mean ± SD for B-D. *p value*: unpaired two tailed t test performed in all comparisons.

We developed a protocol that allowed us to measure directly Ca^2+^ uptake by the store where the SERCA is localized (ER), based in the compartmentation of a fluorescent dye in permeabilized cells. We used the low affinity indicator MagFluo4 which has a Kd for Ca^2+^ of 22 μM (Rossi and Taylor, 2020). The cytosolic concentration of Ca^2+^ in *T. gondii* is around ~70 nM (Moreno and Zhong, 1996), which is considerably below the detection limit of MagFluo4. We loaded parasites for an extended length of time and using high concentrations of MagFluo4-AM as the intention was for the indicator to compartmentalize. At the end of the incubation, parasites were washed and exposed to low concentration of digitonin, which permeabilized the plasma membrane leaving the organellar membranes intact. Under these conditions we had parasites loaded with a Ca^2+^ indicator inside organelles (Fig. 2A). We next determined the capacity of these permeabilized parasites to take up Ca^2+^. SERCA activity is dependent on the presence of Mg-ATP (Fig. 2B) and we added this substrate under defined concentrations of Ca^2+^ (determined using the MaxChelator program). (Bers et al., 1994). We observed that under these conditions a consistent and reproducible Ca^2+^ uptake was measured that was optimal at 220 nM Ca^2+^ and 500 μM MgATP (Figs 2B–C). Validation that this activity was due to SERCA is shown in the experiment in which we added TG highlighting the ER Ca^2+^ leakage (Fig. 2D). Note that the Ca^2+^ released by TG appears to be modest but the Kd of the indicator is 22 μM so a small decrease is highly significant when considering the amount of Ca^2+^ that it represents. Ionomycin can be more effective and larger amounts of Ca^2+^ were released from the ER and other stores as the minimum reached is lower than the starting level of Ca^2+^ (Fig. 2E).

In summary, these results showed that we can measure SERCA activity *in situ* in permeabilized parasites with the low affinity Ca^2+^ indicator MagFluo4. This activity is dependent on MgATP and TG causes release of the accumulated Ca^2+^. The better effect of IO could be due to Ca^2+^ release from other compartments driven by other ATPases (TgA1).

### TgSERCA and the *T. gondii* lytic cycle

**Figure 3:**
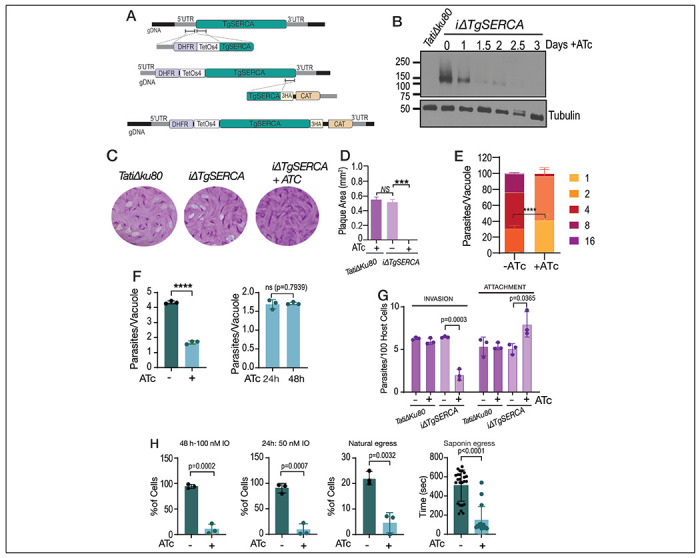
The sarcoplasmic-endoplasmic reticulum calcium ATPase (SERCA) is essential for *T. gondii* replication. **A**, Scheme showing the strategy used for the down regulation of the expression of TgSERCA by promoter insertion and regulation by 0.5 μg/ml Anhydrotetracyclin (ATc). The mutants generated were termed *iΔTgSERCA* or *iΔTgSERCA-HA* for the C-terminal tagged one. DHFR, Dihydrofolate reductase gene for selection with pyrimethamine; CAT, chloramphenicol acetyl transferase gene for selection with chloramphenicol. **B**, Western blots of *iΔTgSERCA-HA* lysates grown with and without ATc. The expression of TgSERCA is shown by the signal obtained with the anti-HA antibody. **C**, plaque assays comparing the size of plaques formed by tachyzoites (150/well) of the *iΔTgSERCA* mutant grown with and without 0.5 μg/ml ATc for 8 days. Plaques formed by the parental strain *TatiΔku80* are shown for comparison. **D**, quantification of the size of the plaques presented in C. **E**, replication assays using the *iΔTgSERCA_RFP* mutant. Number of parasites per parasitophorous vacuole (PV) obtained 24 h after infection of fibroblast cells is reported and compared between the mutant grown with and without 0.5 μg/ml ATc. Data represented as mean ± SD. *p value*: unpaired two tailed t test performed in all comparisons. **F**, Average number of parasites per PV counted at 24 h after the initial infection. The graph shown to the right shows the number of parasites per PV of the *iΔTgSERCA* (+ATc) for 24 or 48 hours after the initial infection. **G**, Invasion assays with the *iΔTgSERCA* mutant treated with ATc for 24 h were done using the red-green assay. **H**, Egress assays with the *iΔTgSERCA* mutant previously grown with or without ATc. Egress was triggered with IO (100 or 50 nM) or saponin (0.01%). The protocol for natural egress uses compound 1 and it is detailed in the methods section.

To investigate the role of TgSERCA (TGGT1_230420) in the biology of *T. gondii* we created conditional KOs (*iΔTgserca*) by inserting a tetracycline regulatable element at the 5’ end of the gene to control the expression of the gene with ATc (Sheiner et al., 2011). In addition, we endogenously tagged TgSERCA with HA. We isolated clonal cell lines of *iΔTgserca* and clones expressing *iΔTgserca-HA* (Fig. 3A). Western blots (Fig. 3B) and immunofluorescence analysis (**Fig. S2**) showed that the expression of TgSERCA is tightly regulated by ATc and it is absent after 1.5 days in culture. The growth of the *iΔTgserca* was severely retarded when cultured in the presence of ATc as evaluated by plaque assays (Fig. 3C–D). In this assay, parasites engage in repetitive cycles of invasion, replication, and egress causing host cell lysis and formation of plaques in confluent monolayers infected with *T. gondii*. Replication was severely impacted after down regulation of SERCA, and parasites were unable to proceed after one or two replication cycles (Fig 3E–F). All (100%) PVs of the ATc cultured mutant contained 4 parasites or less (Fig. 3F, *bar graph on the right*). Invasion of host cells was also reduced in the *iΔTgserca* (+ATc) mutant (Fig. 3G) and egress stimulated by ionomycin, or natural egress followed pre-incubation with Compound 1, was significantly decreased. Interestingly, when testing egress after the addition of saponin in the presence of extracellular Ca^2+^, we observed that the tachyzoites egressed sooner (Fig. 3H, *saponin egress*). This could be because as the SERCA is not active, the cytosolic Ca^2+^ increase due to Ca^2+^ influx from the extracellular milieu after saponin permeabilization cannot be compensated. In addition, the natural efflux from the ER would result in a higher cytosolic Ca^2+^ increase that would cause a faster egress.

### Ca^2+^ uptake by the SERCA-ATPase is essential for filling acidic Ca^2+^ stores

**Figure 4:**
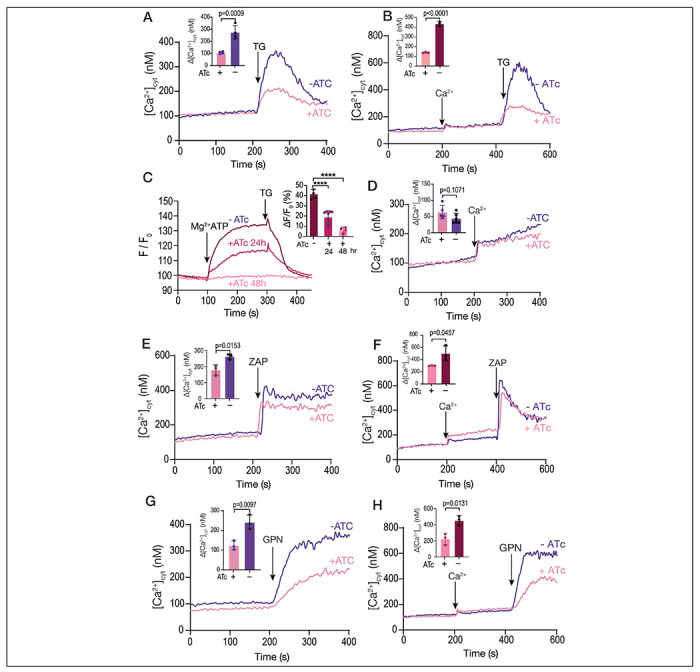
Organellar calcium pools in the *iΔTgserca* mutant. **A**, cytosolic calcium response to the addition of 1 μM TG. The *iΔTgserca* grown with and without ATc was previously loaded with Fura-2 and the cytosolic Ca^2+^ responses were measured. 1 μM TG was added at 200 sec. The purple trace shows the response of the parental cell line grown without ATc and the pink one shows the response of the same mutant grown with ATc for 24 h. The bar graph shows the analysis of the Δ[Ca^2+^] from three independent experiments. **B**, same experimental set-up as the one in A but adding 1.8 mM extracellular Ca^2+^ at 200 sec. **C**, SERCA activity measured with MagFluo4-loaded tachyzoites of *iΔTgserca* mutant previously grown with or without ATc. Parasites were collected and loaded with MagFluo4-AM as described in the Methods section. Digitonin permeabilized parasites were used in the experiment. The concentration of free calcium in the buffer is 220 nM and MgATP (0.125 mM) was added at 100 sec. The response of the control mutant grown without ATc is shown in purple. The other traces show the response of the mutant grown for either 24 or 48 h with ATc. TG was added at 1 μM TG. The bar graph shows the quantification of the slope after adding MgATP. **D**. Ca^2+^ entry measured in Fura-2 loaded *iΔTgSERCA* (±ATc). 1.8 mM extracellular Ca^2+^ was added at 200 sec. The inset shows the ΔF measurement from three independent experiments and showed no significant difference. **E**, Similar conditions to the ones used in A but adding 100 ΔM Zaprinast. The bar graph shows the analysis of the Δ[Ca^2+^] from three independent experiments. **F**, Similar conditions to the ones used in B but adding 1.8 mM extracellular calcium at 200 sec and 100 μM Zaprinast at 400 sec. The bar graph shows the analysis of the Δ[Ca^2+^] from 3 independent experiments. **G**, Same as A but adding 40 μM GPN. The bar graph shows the analysis of the Δ[Ca^2+^] from three independent experiments. **H**, Same set-up as in F but adding 1.8 mM Ca^2+^ at 200 sec followed by 40 μM GPN. The bar graph shows the analysis of the Δ[Ca^2+^] from three independent experiments. Data are presented as mean ± SD. *p* value: unpaired two tailed t test performed in all comparisons.

The *iΔTgserca* mutant showed a low response to TG (Fig. 4A), when measuring cytosolic calcium (Fig. 4A) which was also the case when adding TG after extracellular calcium to fill the store (Fig. 4B). This was most likely the result of the lower activity of TgSERCA. Note that the change in Ca^2+^ in Fig. 4A (*TatiΔku80* cells) is larger than in Fig. 1B (RH strain) and we believe that the difference is related to the characteristics of each cell line (RH vs TatiΔku80). In addition, Mg-ATP-driven Ca^2+^ uptake by permeabilized cells measured with MagFluo4 showed no TgSERCA activity after 48 h of culture with ATc (Fig. 4C). This experiment validated the MagFluo4 approach for determination of SERCA activity. At 24 hours after culturing the parasites with ATc there was still some SERCA activity (Fig. 4C). Ca^2+^ entry measured in Fura2 loaded *iΔTgserca* (±ATc) parasites was not affected by the downregulation of TgSERCA (Fig. 4D). Interestingly, the cytosolic resting Ca^2+^ concentration was not affected in the *iΔTgserca* (+ATc) mutant (Fig. 4A,B, D–H) pointing to a critical role of the plasma membrane Ca^2+^ pump in the homeostasis of cytosolic Ca^2+^. The response to Zaprinast was diminished but was still present (Fig 4E) indicating that Zaprinast acts by releasing Ca^2+^ from the ER and from an additional compartment. Adding Zaprinast after calcium also resulted in a reduced response in the mutant pre-incubated with ATc (Fig 1F). We next tested GPN, which acts mostly on acidic stores and the response was also decreased (Fig. 4G). Adding GPN after replenishing the cells with Ca^2+^ resulted in an increased response as we showed in Fig. 1, but this response was also reduced when the mutant was grown with ATc (Fig. 1H). These results support a link between the stores on which GPN is acting upon and the ER. Considering that downregulation of SERCA impacted the ER storage of Ca^2+^ but did not affect cytosolic Ca^2+^ uptake, the diminished response to GPN, supports that the Ca^2+^ released/leaked from the ER plays an important role in filling the GPN’s targeted store.

### The mitochondrion takes up Ca^2+^ from the ER

**Figure 5:**
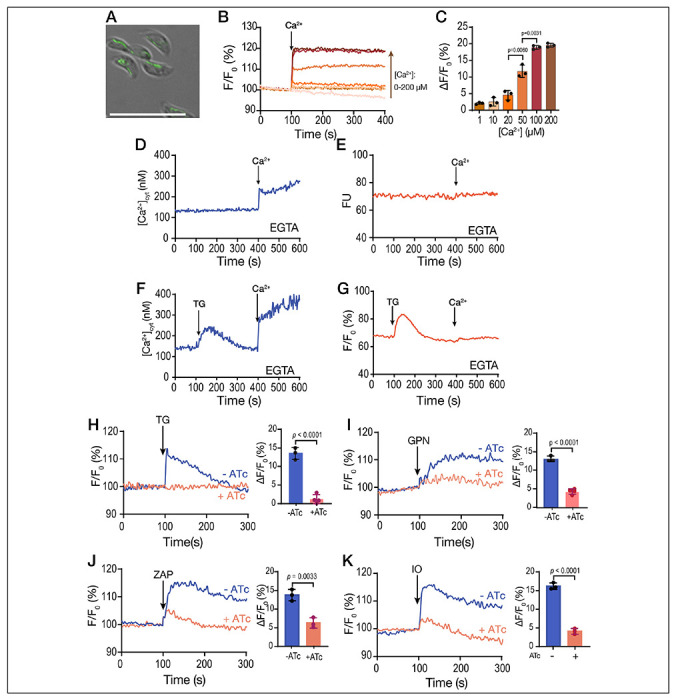
Mitochondrial Calcium uptake. **A**, Fluorescence image of *T. gondii* tachyzoites expressing *SOD2-GCaMP6f* (pDT7S4H3-SOD2-GCaMP6f). The generation of this cell line is described in material and methods. **B**, *T. gondii* tachyzoites expressing SOD2-GCaMP6f (5x10^7^) permeabilized with digitonin as described in the method’s section were tested in a buffer with 100 *μ*M EGTA. Ca^2+^ was added at 100 sec at the final concentrations 1, 10, 20, 50, 100 and 200 *μ*M. Maxchelator was used to calculate the amount of Calcium to be added to attain the indicated concentrations. **C**, ΔF was measured as the change in fluorescence between the baseline and the maximum obtained 20 sec after the addition of Ca^2+^ from three independent biological experiments. **D**, Fura-2 loaded tachyzoites of the SOD2-GCaMP6f expressing mutant in suspension. The experimental set-up was identical to the one described in Fig. 1A–B. 1.8 mM CaCl_2_ was added at 400 sec. Fluorescence measurements were done using the conditions for Fura-2. **E**, GCaMP6 fluorescence measurements of parasites expression SOD2-GCaMP6f in their mitochondrion. The fluorometer conditions were set to measure GCaMP6. **F**, 1 μM TG was added at 100 sec followed by 1.8 mM CaCl_2_ at 400 sec. Fura2 loaded parasites and Fura2 conditions were used. **G**, Same additions as in F but measuring fluorescence of GCaMP6. **H**, Response to the addition of 1 μM TG of the *iΔTgserca-sod2-gcamp6f* mutant (transfected with pCTH3-SOD2-GCaMP6f plasmid) previously grown in the presence (*pink trace*) or absence (*blue trace*) of ATc. The conditions for the fluorescence measurements are the same used in part G with intact parasites. The bar graph shows the ΔF measurements from three independent experiments. **I**, same as H but using 40 μM GPN. **J**, Same as H but using 100 μM Zaprinast. **K**, Same as H but using 1 μM Ionomycin. Data are presented as mean ± SD from 3 independent biological experiments. *p value*: unpaired two tailed t test performed in all comparisons.

In mammalian cells, the high concentration of Ca^2+^ in the ER is important for the production of mitochondrial ATP (Wenzel et al., 2022). This is because of the close proximity between the ER and mitochondria which allows for the directional flow of Ca^2+^ from the ER to the mitochondria (Gincel et al., 2001; Rapizzi et al., 2002). With the aim of determining if the *T. gondii* mitochondria can take up calcium, we introduced a genetic Ca^2+^ indicator in the mitochondrion of *T. gondii* tachyzoites by attaching the *GCaMP6* gene (Chen et al., 2013) to the mitochondrial targeting signal of the *T. gondii* superoxide dismutase 2 (SOD2) gene (Pino et al., 2007) and isolated parasite clones (*Tgsod2gcamp6*) (Vella et al., 2020). GCaMP6 was localized to the mitochondria as detected by Fluorescence (Fig 5A). Direct Ca^2+^ uptake was observed in digitonin permeabilized parasites incubated in the presence of increasing concentrations of Ca^2+^ (Fig 5B–C). A measurable increase was observed but very high concentrations of Ca^2+^ were needed (Fig. 5B–C) indicating that the *T. gondii* mitochondria can take up Ca^2+^ but with very low affinity and that the concentrations of Ca^2+^ needed for the uptake were much higher than the concentrations of cytosolic Ca^2+^ that are present in the cytosol of a live cell (Fig 5B).

We reasoned that the mitochondrion could be taking up Ca^2+^ through close membrane contacts with the ER as it is possible that the concentration of Ca^2+^ in those microdomains could reach much higher levels than in the cytosol and would allow for mitochondrial uptake. We next loaded the *Tgsod2gcamp6* mutant with Fura2 and measured, first, if adding extracellular Ca^2+^ to the parasite suspension led to an increase in GCaMP6 fluorescence. Parallel experiments looking at cytosolic and mitochondrial Ca^2+^ showed that adding extracellular Ca^2+^ does result in an increase in the cytosol, however mitochondrial Ca^2+^ was not affected (Fig. 5D–E). We next added TG followed by Ca^2+^ and this led to an increase in cytosolic Ca^2+^ but only TG resulted in a measurable increase in fluorescence of the mitochondrial indicator (Fig 5G). Our interpretation is that after adding TG, there was accumulation of Ca^2+^ at the outer side of the ER membrane leading to an increased concentration of the ion which activated mitochondrial uptake.

We next inserted the *SOD2GCaMP6* chimeric gene in the *iΔTgserca* mutant and isolated the clonal mutant *iΔTgserca-sod2gcamp6*. We first looked at mitochondrial uptake after adding TG and as shown previously, the parasites cultured without ATc showed a measurable and consistent increase in GCaMP fluorescence after adding TG (Fig. 5H, *blue trace*). This was abolished in cells cultured in the presence of ATc as the low expression of TgSERCA would result in the ER depletion of Ca^2+^ (Fig. 5H, *pink trace*). Interestingly, we observed an increase in the GCaMP fluorescence after adding GPN, ionomycin or Zaprinast (Fig. 5H–J, *blue traces*). In all cases the fluorescence increase was muted in the *iΔTgserca* (+ATc) and the difference in the response was always significant (Fig. 5H–J, *pink traces*).

In summary, we demonstrated that the mitochondrion of *T. gondii* can take up Ca^2+^ through its transfer from the ER, a phenomenon that becomes evident after blocking the ER uptake with TG as the concentration of Ca^2+^ needed to activate the uptake is very high and only reachable at membrane contact sites between the ER and mitochondria. The *iΔTgserca* (+ATc) mutant, is unable to keep the ER filled with Ca^2+^ and this impacts the capacity of the mitochondria to take up Ca^2+^. The mitochondria could be also taking up Ca^2+^ directly from acidic stores, which became depleted when TgSERCA was downregulated.

### Interaction between the ER and the mitochondrion and acidic compartments.

**Figure 6:**
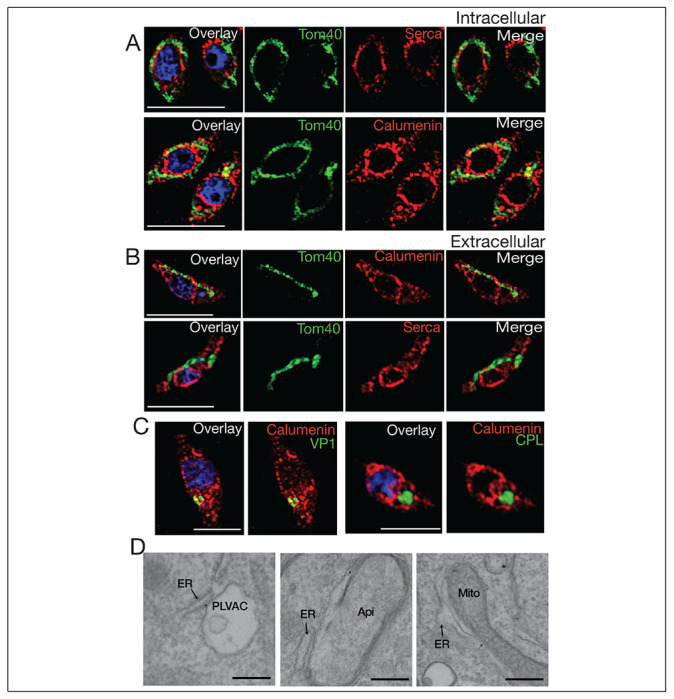
ER membrane contacts with the mitochondrion and the plant like vacuolar compartment (PLVAC). **A**, Super-resolution IFAs of intracellular parasites with the mitochondrion labeled with the αTom40 (green, 1:20,000) antibody and the ER labeled with the αTgcalumenin antibody (an ER calcium binding protein) (red, 1:1000) or the TgSERCA (red 1:1000). **B**, Immunofluorescence (IFA) of extracellular tachyzoites with the same antibodies used for part A. The mitochondrion and ER membranes interact in several regions. **C**, The PLVAC was localized with the αVP1 antibody (green, 1:200) or the αTgCPL antibody (green, 1:500). The ER membranes were labeled with the αTgERC antibody (red). The spots of contact between the ER and the PLVAC are yellow. Scale bars in A-C represent 5 μm. **D**, Transmission Electron Microscopy imaging of the contact sites formed between ER and PLVAC, ER and Apicoplast, ER and mitochondrial. Size bars are 100 nm.

We next investigated if it was possible to see interaction between the ER with other organelles by IFA and/or EM. We performed IFAs with ER and mitochondria markers and ER and PLVAC markers (Fig. 6). We observed that in intracellular parasites the mitochondrion surrounds the ER and multiple potential points of interaction are seen (Fig. 6A and supplemental video 1). The mitochondrion of intracellular *T. gondii* tachyzoites surrounds the periphery of the cell in a lasso-shape morphology. In extracellular parasites the mitochondrion changes drastically its shape adopting either a sperm-like or a collapsed conformation (Ovciarikova et al., 2017) (Fig. 6B and supplemental video 2). Our hypothesis is that this retraction of the mitochondrion allows the ER to expand in extracellular parasites into ER tubules, which extend toward the apical domain of the parasite where Ca^2+^ is needed for secretion of micronemes and conoid extrusion.

The PLVAC also showed points of contact with the ER (Fig. 6C and supplemental video 3). Multiple points of contact were also observed by EM between the ER and the PLVAC, the ER and the apicoplast and the ER and the mitochondrion (Fig. 6D).

Interestingly these contacts were still present in the *iΔTgserca* (+ATc) mutant (**Fig. S3, A-D**), as most likely TgSERCA would not be directly involved in the establishment of contacts. We quantified the length of the limiting membrane of the organelle in contact with ER tubules at a distance of less than 30 nM and found that after knockdown of TgSERCA, the contact was not altered. However, Calcium transported from the ER into the mitochondrion after thapsigargin treatment was significantly decreased which means that this phenotype is due to reduced ER Calcium and not to lack of contacts (**Fig. S3E**). A similar result was seen when measuring contacts between the ER and the apicoplast (**Fig. S3F**) and between the ER and the PLVAC (**Fig. S3G**).

In summary, this data showed that the ER form contacts with other organelles like the mitochondrion, the PLVAC and the apicoplast and these contacts could be the ones allowing the transfer of Calcium from the ER, the store with the highest amount of Calcium, to the other organelles. The SERCA high affinity for Calcium is critical for the filling of the ER. However, the molecular mechanisms involved in the uptake by the other organelles is less clear. Very little is known about the structure and composition of Membrane Contact Sites in *T. gondii* (Huet and Moreno, 2023). However, functional evidence for the transfer of Calcium has been demonstrated for the ER-apicoplast (Li et al., 2021) and in this work we presented evidence for the transfer of Calcium between the ER and the mitochondrion and the ER and the PLVAC.

## DISCUSSION

In this work we showed that the endoplasmic reticulum (ER) of *T. gondii* has an impressive capacity for capturing Ca^2+^ that enters the cytosol from the extracellular milieu, and it accomplishes this with a minimal increase in cytosolic Ca^2+^ levels. This is the result of a highly efficient SERCA Ca^2+-^ATPase (TgSERCA) which also showed high affinity for Ca^2+^. The TgSERCA activity combined with the plasma membrane Ca^2+^ pump (Luo et al., 2001; Luo et al., 2005) would not allow the cytosolic Ca^2+^ to increase above 200 nM (Hortua Triana et al., 2024).

We also provide evidence of the ER’s capacity for sharing Ca^2+^ with other organelles, which originates from the ER unique ability to capture a sizable portion of extracellular Ca^2+^. We showed that extracellular Ca^2+^ can be efficiently taken up into the ER through the activity of SERCA and from the ER it can be transferred to other compartments like the mitochondria and acidic stores. These activities would ensure that Ca^2+^ is released at specific locations without flooding the whole cell with Ca^2+^, which may trigger unnecessary signals. Although initial data suggested a less important role for the mitochondria and PLVAC in Ca^2+^ storage, our results showed that these organelles may become more relevant Ca^2+^ reservoirs during ER calcium overload

The SERCA Ca^2+^-ATPase is a P-type ATPase found in the membranes of the ER and the secretory pathway (Kuhlbrandt, 2004). The mammalian genome encodes for three SERCA pumps: SERCA1, SERCA2, and SERCA3, and seven different isoforms expressed from these genes, SERCA1a/1b, SERCA2a/2b, and SERCA3a/3b/3c (Periasamy and Kalyanasundaram, 2007). SERCA2b is the most widespread of all isoforms and phylogenetically likely the oldest and considered the house-keeping isoform (Wuytack et al., 2002). SERCA pumps translocate two Ca^2+^ ions and hydrolyze one ATP for each catalytic turnover. This pumping activity serves several roles. By translocating Ca^2+^ from the cytosol into the lumen of the ER, it restores the cytosolic Ca^2+^ concentration to its low resting level (<100 nM). The stored Ca^2+^ can be released and activate many cellular activities. In addition, SERCA maintains a sufficiently high (~500 μM) luminal Ca^2+^ concentration that is necessary for enzyme activities important for proliferation, growth, and differentiation (Wuytack et al., 2002).

*T. gondii* appears to express a single SERCA protein (TgSERCA) (Nagamune and Sibley, 2006), most likely playing a housekeeping role like the mammalian SERCA2. This was evident from the strong phenotype of the *iΔTgserca* (+ATc) mutant, which could not replicate after 24 h and was defective for all the steps of the tachyzoite lytic cycle. The activity of TgSERCA was highly dependent on the presence of its substrate MgATP and it showed high affinity for Ca^2+^. Considering the low affinity of the indicator Magfluo4 (Kd: 22 μM), the measured uptake activity and the accumulation of Ca^2+^ was impressive as it was evident even at 55 nM free calcium. This result signified that TgSERCA is highly efficient at keeping the cytosol at the low 60-100 nM level as well as efficiently filling the ER with Ca^2+^.

Ca^2+^ signals are specifically contingent on their spatial, temporal and amplitude features. Movement of Ca^2+^ in the cytosol of cells is severely limited due to the presence of high affinity Ca^2+^ buffers. The concept of Ca^2+^ tunneling through the ER proposed that Ca^2+^ would move more easily within the lumen of the ER as its binding capacity is almost 100 lower than the binding capacity of the cytosol (Mogami et al., 1997) and the high mobility of Ca^2+^ through the lumen of the ER was directly demonstrated (Park et al., 2000), and especially important in polarized cells. In addition, the ER facilitates Ca^2+^ tunneling through its lumen as a mechanism of delivering Ca^2+^ to targeted sites without activating inappropriate processes in the cell cytosol (Petersen et al., 2017). In *T. gondii*, the Ca^2+^ binding capacity of the cytosol compared to the ER is less known as the localization of all its predicted Ca^2+^ binding proteins have not been completely determined and several calmodulin-like proteins showed localization to the conoid (Long et al., 2017).

In *T. gondii* cytoplasmic Ca^2+^ increases, due to efflux from the ER, have been reported to initiate essential parasite traits like microneme secretion (Carruthers and Sibley, 1999; Nagamune et al., 2007b), conoid extrusion (Del Carmen et al., 2009), invasion (Vieira and Moreno, 2000; Lovett and Sibley, 2003) and egress (Arrizabalaga et al., 2004). These responses require precise spatiotemporal control of diverse targets and suggests the presence of distinct systems to deliver Ca^2+^ to specific locations. Our hypothesis is that the ER plays a central role by distributing Ca^2+^ to those locations at specific times for the initiation of parasite functions. The strong invasion, replication and egress phenotypes of the *iΔTgserca* (+ATc) supports this hypothesis.

In mammalian cells Ca^2+^ ion is transferred from the ER to the mitochondrion through the outer membrane voltage-dependent anion channel1 (VDAC1) (Gincel et al., 2001; Rapizzi et al., 2002) and the inner membrane calcium uniporter (MCU1) (De Stefani et al., 2011). A VDAC homologue is present in *T. gondii*, which was shown to be essential for growth and for mitochondrial and ER morphology (Mallo et al., 2021). However, the molecular evidence for the presence of a Ca^2+^ uniporter in the inner mitochondrial membrane driven by the electrochemical gradient generated by the electron transport is not supported by the genome database. Calcium transport into the *T. gondii* mitochondrion had not been previously shown and our data represents the first experimental evidence showing this event although the mechanism is less clear. We showed that it has very low affinity for Ca^2+^ and it was only possible to observe it in intact parasites after inhibiting SERCA, which allowed the increase of the concentration of Ca^2+^ at the local ER membrane. Over the past decade a new concept about the communication between subcellular organelles through tight, stable, and yet non-fusogenic associations known as membrane contact sites (MCSs) has emerged (Phillips and Voeltz, 2016). The ER forms an extensive network of MCSs with the PM, mitochondria and endocytic vesicles for the exchange of Ca^2+^ (Burgoyne et al., 2015).

In *T. gondii* the characterization of MCSs is only in its beginnings (Huet and Moreno, 2023) with only a few evidences for their presence (Tomova et al., 2009; Mallo et al., 2021; Ovciarikova et al., 2022) and function (Li et al., 2021; Oliveira Souza et al., 2022). When *T. gondii* is intracellular, microscopy observations showed that its mitochondrion surrounds the periphery of the cell in a lasso-shape conformation. On the other hand, in extracellular parasites the mitochondrion changes its morphology and adopts a sperm-like or collapsed conformation (Ovciarikova et al., 2017). Our IFA analysis with ER and mitochondrial markers showed that the lasso-shaped mitochondrion surrounds the ER with plenty of opportunities for contact between both organelles. We speculate that at this stage the mitochondrion is buffering any leakage of Ca^2+^ from the ER to prevent premature egress as a cytosolic increase would stimulate motility and possibly egress.

**Figure 7:**
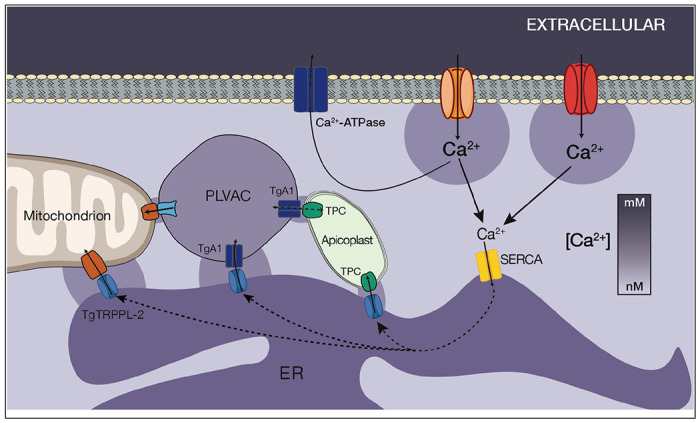
Model showing calcium entry through two different types of Ca^2+^ channels, uptake to the ER by SERCA and distribution to the other organelles via transfer from the ER to the mitochondria, PLVAC and apicoplast. In intracellular parasites, the mitochondria is in close contact with the ER which is constitutively leaking Ca^2+^ into the cytosol. The mitochondria take up Calcium from the ER through an unknown mechanism. VDAC could be involved in the transfer through the outer mitochondrial membrane (Mallo et al., 2021). The PLVAC interacts with the ER and may also interact with the mitochondrion and the apicoplast. TgA1, a calcium ATPase previously characterized may be the pump involved in uptake (Luo et al., 2001; Luo et al., 2005). The mechanism of release is unknown. The TPC was shown to be involved in the formation of contacts between the ER and the apicoplast (Li et al., 2021). Ca^2+^ could leak from the ER through the TgTRPPL-2 channel previously described (Marquez-Nogueras et al., 2021).

In this work, we showed that Ca^2+^ can be transferred from the ER to the mitochondrion, most likely through MCSs as the low affinity of the mitochondrion would not permit uptake directly from the cytosol. Down regulation of TgSERCA resulted in diminished response to acidic stores triggers, which supported that the ER may also be responsible for their filling. The mitochondrion may also be able to take up Ca^2+^ from acidic stores like the PLVAC as GPN was also able to stimulate Ca^2+^ uptake and the response was diminished in the *iΔTgserca* (+ATc) mutant. Transfer of Ca^2+^ from acidic stores to the mitochondrion was impacted by the ER depletion (Fig. 7).

In conclusion, this study showed how the ER of *T. gondii* replenish itself with calcium and how it supplies Ca^2+^ to the cytosol for signaling and to acidic stores and the mitochondrion. *T. gondii* is a protozoan parasite that causes disease by reiterating a lytic cycle that is driven by calcium signaling. This work contributes to the understanding of how extracellular and intracellular Ca^2+^ stores work together to sustain the pathologic features of *T. gondii*. Future studies would aim at finding more about the role of Ca^2+^ in mitochondrial and acidic stores functions.

## Methods

### Cell Culture—

*Toxoplasma gondii* tachyzoites (RH strain) were maintained in human telomerase reverse transcriptase immortalized foreskin fibroblasts (hTERT) (Farwell et al., 2000) grown in Dulbecco’s modified minimal essential media (DMEM) with 1% FBS.

### Generation of SERCA mutants—

A promoter insertion plasmid was generated by cloning three PCR fragments into a modified pCR2.1-TOPO vector using Gibson Assembly Cloning Kit (NEB #E5510). One fragment corresponding to the TgSERCA flanking region (predicted promoter/5’UTR) was amplified with primers 1 and 2 (Table S1). The second fragment corresponds to DHFR+T7S4 (Sheiner et al., 2011) and was amplified with primers 3 and 4. Another fragment corresponds to the 5’ TgSERCA coding sequence beginning with start codon and was amplified with primers 5 and 6. The vector pCR2.1-TOPO was used, which had only one EcoRI site and it was cut with the enzyme NotI to use as a vector backbone. The promoter insertion plasmid was transfected into the *TatiΔku80* cells and selected with 1 μM pyrimethamine using a “ultra aggressive” screening method. Briefly, 200 μl of the suspension of transfected parasites was added to 10 ml of media and then one to three drops (~65 μl per drop) were inoculated into three 24-well plates containing media. The clonal lines created after selection and subcloning were termed *iΔSERCA*.

For *in situ* tagging, an approximately 2 kb fragment was amplified from the genomic locus (3’ region) of the TgSERCA gene using primers 7 and 8. The fragment was cloned into the pLic-3HA-CAT plasmid (Huynh and Carruthers, 2009) and the construct was linearized with the enzyme NheI for transfection into the *iΔSERCA* mutant. Clonal cell lines were generated after selection with chloramphenicol and subcloning and termed *iΔSERCA-3HA*.

### Expression and purification of TgSERCA recombinant protein—

The phosphorylation(P) and nucleotide binding(N) domains of TgSERCA (TGGT1_230420) (nucleotides 1123 to 2415, amino acid residues 375 to 805) were cloned into XmaI and HindIII sites of pQE-80L with primers 13 and 14 to create recombinant protein with a N-terminal 6xHis tag. The resulting plasmid was transformed into *Escherichia coli* BL21-CodonPlus competent cells and expression was induced by addition of 0.4 mM isopropyl β-D-1-thiogalactopyranoside (IPTG) for 4 hours at 37°C. Cells were pelleted and resuspended in equilibration/binding buffer (50 mM Na_3_PO_4_, 300 mM NaCl, 10 mM Imidazole, 8 M Urea, and protease inhibitor cocktail P-8849). The cells were then sonicated for 80 seconds total and centrifuged at 12,000 rpm for 20 mins at 4°C. The supernatant was filtered through a 0.45 μm membrane and the protein was purified using HisPur Ni-NTA Chromatography Cartridge (Thermo Scientific) following instructions from the manufacturer. Proteins that were unbound were washed off with 12 ml of wash buffer (50 mM Na_3_PO_4_, 300 mM NaCl, 40 mM imidizole, and 8 M urea), and the recombinant protein was eluted with 5 ml elution buffer (50 mM Na_3_PO_4_ , 300 mM NaCl, 250 mM iImidizole, and 8 M urea). Eluted protein fractions were concentrated and desalted with Amicon Ultra-0.5 mL centrifugal filter (Millipore Sigma).

### Anti-TgSERCA antibody generation in Guinea pig—

Two Guinea pigs were each immunized with 0.2 mg of purified recombinant protein mixed with equal volume of Freund’s Complete Adjuvant (Sigma F5581), followed by two boosts of 0.1 mg antigen mixed with equal volume of Freund’s Incomplete Adjuvant (Sigma F5506) for Guinea pig 1 and three boosts for Guinea pig 2. The resulting antibodies were tested at 1:1000 in western blot against RH lysates and were developed with Alexa Fluor 488 goat anti-Guinea pig (1:1000). The antibodies were compared with Dr. Sibley’s mouse anti-SERCA antibody (Nagamune et al., 2007a) to confirm size and purity. The anti-*Tg*SERCA antibodies were then affinity purified.

### Cytosolic calcium measurements with Fura-2—

*T. gondii* tachyzoites were loaded with Fura-2 AM as previously described (Vella et al., 2020; Stasic et al., 2021). Freshly released tachyzoites were washed twice with buffer A plus glucose (BAG; 116 mM NaCl, 5.4 mM KCl, 0.8 mM MgSO_4_, 50 mM Hepes, pH 7.3, and 5.5 mM glucose), by centrifugation (706 x *g* for 10 min) and re-suspended to a final density of 1 x l0^9^ parasites/ml in loading buffer (BAG plus 1.5% sucrose, and 5 μM Fura2 AM). The suspension was incubated for 26 min at 26 °C with mild agitation. Subsequently, the parasites were washed twice (2000 x *g* for 2 min) with BAG to remove extracellular dye, re-suspended to a final density of 1x10^9^ parasites per ml in BAG and kept on ice. For fluorescence measurements, 2 x 10^7^ parasites/mL were placed in a cuvette with 2.5 mL of Ringer’s buffer without calcium (155 mM NaCl, 3 mM KCl, 1 mM MgCl_2_, 3 mM NaH_2_PO_4_, and 10 mM Hepes, and 10 mM glucose). Fluorescence measurements were done in a Hitachi F-7000 fluorescence spectrofluorometer using the Fura-2 conditions for excitation (340 and 380 nm) and emission (510 nm). The Fura-2 fluorescence response to Ca^2+^ was calibrated from the ratio of 340/380 nm fluorescence values after subtraction of the background fluorescence of the cells at 340 and 380 nm as previously described (Grynkiewicz et al., 1985). The Ca^2+^ release rate was defined as the change in Ca^2+^ concentration during the initial 20 s after reagent addition. Delta F was calculated by the difference between the higher calcium peak and basal Ca^2+^, and recovery was defined as the change of Ca^2+^ concentration after the calcium peak was reached for the subsequent indicated times.

### Endoplasmic reticulum Ca^2+^ Measurements in permeabilized T. gondii tachyzoites—

Tachyzoites freshly egressed and washed as described above were resuspend to a final density of 1x10^9^ cells/ml in HBS buffer (135 mM NaCl, 5.9 mM KCl, 1.2 mM MgCl_2_, 11.6 mM HEPES pH 7.3, 1.5 mM CaCl_2_, 11.5 mM glucose) containing 1 mg/ml BSA, 0.2 mg/ml of pluronic F127 and 20 μM MagFluo4/AM. The suspension was incubated in a 20°C water bath with mild shaking for 1 h, in the dark. Subsequently, parasites were washed 3 times at 5,000 rpm for 2 min to remove extracellular dye. The pellet was resuspended in 1.8 ml of CLM buffer (20 mM NaCl, 140 mM KCl, 20 mM PIPES, pH 7.0) containing 1 mM EGTA at 1x10^9^ cells/ml. Cells were permeabilized with 44.4 μg/ml digitonin for 10 min. Cells were washed 3 times with CLM containing 1 mM EGTA at 5,000 rpm for 2 min to remove digitonin and resuspended to a final density of 1x10^9^ tachyzoites/ml and kept in ice. For each test, 50 μl (5x10^7^) of parasite suspension was added to 1.95 ml of CLM containing 1 mM EGTA and 0.375 mM CaCl_2_ which results in 220 nM free Ca^2+^ as calculated with MaxChelator. Fluorescence was measured with an Hitachi F-7000 fluorescence spectrometer (Excitation at 485 nm and emission at 520 nm). Ratio (ΔF/F_0_) was evaluated by measuring the rate of change in fluorescence over 20 s after reagent addition (linear rate).

### Strain construction and maintenance—

The organelle targeting of GCaMP6f was made by overlapping PCR. The N-terminal mitochondrial targeting sequence of the *T. gondii* SOD2 gene (Pino et al., 2007) was used to target GCaMP6f to the mitochondrion. The Gcamp6f for this construct was amplified by primers 9 and 10 (Table S1). After gel purification of the GCaMP6f and SOD2 sequences, the mitochondria targeting construct was built by overlapping PCR with the purified PCR products as template. This construct was then cloned into the Topo-blunt vector. After the sequence was verified by sequencing, the SOD2-Gcamp6f fragment was removed by BglII and AvrII digestion and cloned into the same restriction sites of the pDT7S4H3 (Sheiner et al., 2011) and pCTH3 (Vella et al., 2020) vectors. The pDT7S4H3-SOD2-GCaMP6f construct was introduced into RH parasites by electroporation. After selection with pyrimethamine, the parasites were sorted by FACS and then subcloned. Clones were selected based on the dynamic range of the response to ionomycin. The pCTH3-SOD2-GCaMP6f was introduced into the *iΔTgSERCA* mutant by electroporation. After selection with chloramphenicol, the parasites were sorted by FACS and then subcloned. The expression of GCaMP6f was verified by live cell imaging, IFA, and western blots. The clone with the largest dynamic range, as evaluated with Ionomycin, was selected to further use.

### GCaMP6f fluorescence Measurements—

Permeabilized tachyzoites: *T. gondii* tachyzoites expressing SOD2-GCaMP6 were collected and washed 3 times at 5,000 rpm for 2 min. The parasite pellet was resuspended in 1.8 ml of BAG buffer containing 0.1 mM EGTA at 1x10^9^ cells/ml. Permeabilization with 44.4μg/ml digitonin for 10 min was done by following the fluorescence of GCaMP6. Cells were washed 3 times with the same buffer at 5,000 rpm for 2 min to remove digitonin, resuspended to a final density of 1x10^9^ parasites/ml and kept in ice. 50 μl (5x10^7^) of the parasite suspension was mixed with 1.95 ml reaction buffer containing 0.1 mM EGTA.

For measurements with intact parasites, they were collected, washed, and resuspended in BAG at 1x10^9^ cells/ml for testing. Measurements were done in a Hitachi 7000 fluorescence spectrometer set at excitation, 485 nm and emission, 509 nm. The ratio (ΔF/F_0_) was evaluated by measuring the rate of change in fluorescence over 20 s after reagent addition (linear rate).

### Growth, Invasion and Egress assays—

Red-green invasion assays were performed as originally described (Kafsack et al., 2004), modified (Chasen et al., 2017) and adapted to use td-RFP expressing parasites. The number of tachyzoites used was 2 x 10^7^, and invasion was for 5 min. Plaque assays were performed as described in (Liu et al., 2014) with modifications. 125 tachyzoites were used for infection of confluent six well plates with hTert fibroblasts followed by an incubation time of 10 days prior to fixing and staining with crystal violet.

For egress assays, HeLa cells were infected with either parental or *iΔTgSERCA-HA* tachyzoites. *iΔTgSERCA-HA* tachyzoites were grown in HeLa cells in the absence or presence of anhydrous tetracycline for the duration of the infection. Forty-eight hours after infection, parasitophorous vacuoles containing 4-8 parasites were monitored by microscopy after washing with Ringer buffer. CaCl_2_ was omitted for experiments in the absence of extracellular calcium, and the media was supplemented with 100 μM or 1 mM EGTA to chelate contaminating calcium. For experiments containing CaCl_2_, 2 mM CaCl_2_ was added to the calcium-free Ringer buffer right before the addition of the specific triggers. Drugs were added in calcium free Ringer buffer at the concentrations indicated: ionomycin (1 μM), thapsigargin (1 μM), zaprinast (100 μM), saponin (0.01%). Ringer buffer was used as extracellular buffer (EB).

For natural egress, the *iΔTgSERCA* mutant expressing td-tomato RFP was used to infect confluent host cell monolayers 36 hours before adding ATc and 1 μM compound 1 (pyrrole 4-[2-(4-fluorophenyl)-5-(1-methylpiperidine-4-yl)-1H-pyrrol-3-yl]pyridine) (Donald et al., 2002) dissolved in ethanol and the culture continued for 24 hours. After treating with cpd1 for 24 hours, cultures showed full vacuoles which differed from the vehicle treated plates (36 hours cultured plus ATc treatment for 24 hours or 48 hours without ATc), which were fully lysed. The cpd1 media was removed and the synchronized vacuoles were washed twice, and the media replaced with media without cpd1. The plates were then moved to the Prewarmed DeltaVision microscope for observation. After 10 minutes at 37C, egress of the full vacuoles was enumerated. We counted each plate for 1 minute and we counted at least 100 vacuoles per experiment. 3 independent biological experiments were conducted and summarized.

For replication assays, the hTERT cells were grown on 35 mm Mattek dishes. Each dish was infected with 50,000 tdTomato-expressing parasites. 24 h after the infection the counting of parasites per PV was done in a fluorescence microscope. For each experiment, at least 100 PVs were counted. Results were the average of 3 independents experiments (Li et al., 2021).

### Microscopy and western blot analyses—

Tachyzoites were grown on hTERT cells on cover slips for ~24 hr, washed twice with buffer A with glucose (BAG, 116 mM NaCl, 5.4 mM KCl, 0.8 mM MgSO_4_, 50 mM HEPES, pH 7.2, and 5.5 mM glucose) and fixed with 4% formaldehyde for 1 h, followed by permeabilization with 0.3% Triton X-100 for 20 min, and blocking with 3% bovine serum albumin. IFAs were performed as previously described (Miranda et al., 2010). Fluorescence images were collected with an Olympus IX-71 inverted fluorescence microscope with a Photometrix CoolSnapHQ CCD camera driven by DeltaVision software (Applied Precision, Seattle, WA). Superresolution microscopy was performed using a Zeiss ELYRA S1 (SR-SIM) microscope with a high-resolution Axio observer Z1 inverted microscope stand with transmitted (HAL), UV (HBO,) and high-power solid-state laser illumination sources (405/488/561 nm), a 100x oil immersion objective, and an Andor iXon EM-CCD camera and acquired using the ZEN software (Zeiss) with a SIM analysis module. Rat anti-HA antibody from Roche was used at a dilution of 1:25. Mouse anti-HA antibody from Covance was used at a dilution of 1:200.

Western blot analysis was performed as previously described (Liu et al., 2014). Rat anti-HA antibody from Roche was used at a dilution of 1:200. Mouse anti-HA antibody from Covance was used at a dilution of 1:1,000. Secondary goat anti-rat or mouse antibody conjugated with HRP was used at 1:5,000. Mouse anti-α-tubulin at a dilution of 1:5,000 was used for loading control.

### Transmission Electron Microscopy—

For ultrastructural observations of intracellular *T. gondii* by thin-section transmission electron microscopy (EM), infected human foreskin fibroblast cells were fixed in 2.5% glutaraldehyde in 0.1 mM sodium cacodylate (EMS) and processed as described (Coppens and Joiner, 2003). Ultrathin sections of infected host cells were stained before examination with a Hitachi 7600 EM under 80 kV. For quantitative measurement of distance between organelles, the closest point between *T. gondii’s* organelles and ER membrane was measured using ImageJ and was performed on 47 representative electron micrographs at the same magnification for accurate comparison between organelles

### Statistical Analysis—

Statistical analyses were performed by Student’s t-test using GraphPad PRISM version 9. Error bars shown represent mean ± SD (standard deviation) of at least three independent biological replicates. Unpaired two tailed t test performed in all comparisons.

## HIGHLIGHTS

- The *T. gondii* ER efficiently takes up Ca^2+^ that enters the cytosol from the extracellular milieu.
- Filling of acidic stores in *T. gondii* appears to be dependent on the filling of the ER
- The mitochondrion of *T. gondii* has no direct access to extracellular calcium but is able to take up Ca^2+^ by transfer from the ER and/or acidic stores
