# Supplementary material for: Calcium transfer from the ER to other organelles for optimal signaling in Toxoplasma gondii
Source: bioRxiv. 2024 Dec 5:2024.08.15.608087. Originally published 2024 Aug 15. Preprint. [Version 2] doi: 10.1101/2024.08.15.608087 (PMC11343207; doi:10.1101/2024.08.15.608087)
Supplement: Supplement 1 [file media-1.pdf]

Calcium transfer from the ER to other organelles for optimal signaling in *Toxoplasma gondii*.

Zhu-Hong Li et al.

Supplemental Material

Supplemental Table 1: Primers used in this study

Primer Number	Sequence	Purpose
1: SERCA_5'UTR_F	ttctgcagatatccatcacactggcCCACCTCCTCGGCGGTTCTCTCTCG	Amplify 5'UTR of *TgSERCA*
2: SERCA_5'UTR_R	aggtttcgtgctgCGCTGCGTCTCCGAAGATAAGCCGAAC	
3: DHFR+T7S4_SERCA_F	cggagacgcagcgCAGCACGAAACCTTGCATTCAAACC	Amplify DHFR+T7S4 cassette
4: DHFR+T7S4_SERCA_R	ttgacaggtccatGGTTGAAGACAGACGAAAGCAGTTG	
5: SERCA_3'HR_F	tctgtcttcaaccATGGACCTGTCAAACGAGAAAGCCG	Amplify the 3' homologous region of *TgSERCA* for promoter insertion
6: SERCA_3'HR_R	gggccctctagatgcatgctcgagcGGAGACTCTGAATGAGTGAACACGAG	
7: SERCA-LIC-F	TACTTCCAATCCAATTTAATGCCGACGATCCCTGCTCCTT	Amplify 3' SERCA for LIC into plic-3HA
8: SERCA-LIC-R	TCCTCCACTTCCAATTTTAGCCTGCAGCTTGCGCAGCTG	
9: R-6f-AvrII-NoSC	CCTAGGCTTCGCTGTCATCATTTGTACA	Make SOD2-Gcamp6 construct
10: F-SOD2-BglII	AGATCTATGTCCATCACAGCTGTCCTAGTGCCAG	
11: R-SOD2-4-Gcamp6	TGAGAACCCATGGCGTTTGTGGAGAAACAGTGGGC	Make SOD2-Gcamp6 construct
12: F-Gcamp6-4-SOD2	CCACAAACGCCATGGGTTCTCATCATCATCATCATC	
13: XmaI_SERCA_PNP_F	5' ACGTCCCGGGTGCCATCGTGAGAAAGCTCGCG 3'	Amplify *TgSERCA* to prepare recombinant protein for Ab production
14: HindIII_SERCA_PNP_R:	5' ACGTAAGCTTGTTGTCGTCTGCGAGAACCATG 3'	

Supplemental video legends:

Supplemental Video 1. IFA of intracellular parasites labeled with the mitochondria marker αTom40 (green) antibody, and the ER labeled with the αTgERC antibody (red). Image acquisition using Zeiss Elyra Super resolution microscope and 3D visualizations using Imaris version 10.1.

Supplemental Video 2. Imaris 3D optimal visualization of extracellular parasites labeled with the αTom40 (green) antibody and the ER labeled with the αTgERC antibody (red).

Supplemental Video 3. ER membrane contacts sites with the plant like vacuolar compartment (PLVAC). Imaris 3D visualization of immunofluorescence of extracellular tachyzoites. PLVAC was labeled with αVP1 antibody (green) and ER was labeled with αTgERC antibody (red)

Supplemental Figures

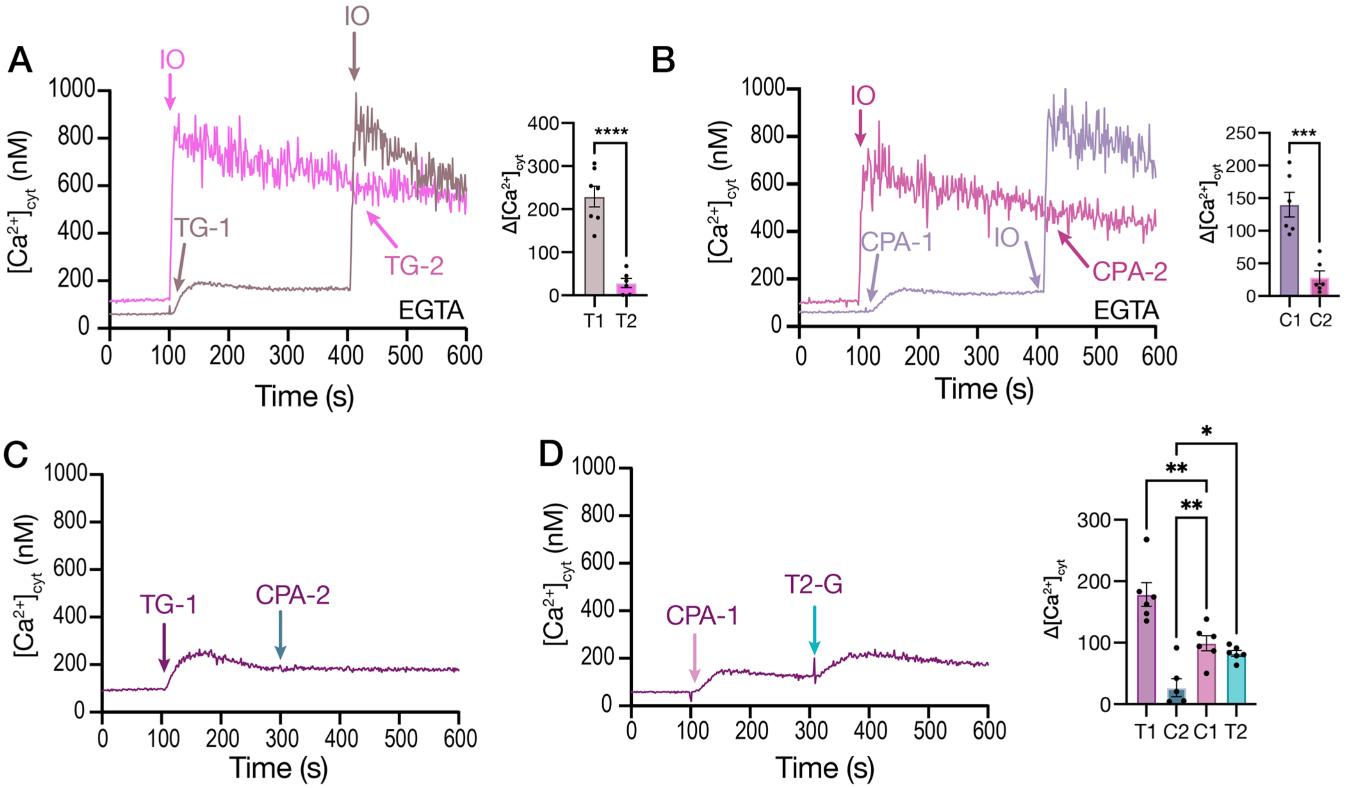

Figure S1: Intracellular Calcium pools. *T. gondii* tachyzoites loaded with Fura2 were used for these measurements. The suspension was in Ringer buffer with 100 μM EGTA. **A**, Thapsigargin (TG), 1 μM was added at 100 seconds (TG-1) followed by Ionomycin 1 μM (*brown trace*). Switched additions, IO 1 μM followed by TG 1 μM is shown in the experiment depicted by the pink trace. The bar graph shows the statistical analysis of $\Delta[Ca^{2+}]$ cytosolic obtained after the additions of TG first (T1) compared with the same addition after IO (T2) obtained from more than three biological experiments. **B**, Similar experiment to the one shown in A but using cyclopiazonic acid (CPA) 10 μM and IO 1 μM. **C**, TG 1 μM followed by CPA. **D**, CPA 10 μM followed by TG 1 μM. The bar graph shows the analysis of the cytosolic $\Delta[Ca^{2+}]$ after the addition of TG (T1 and T2) or CPA (C1 and C2) from more than three biological experiments. Data are presented as mean ± SD. *p value*: unpaired two tailed t test performed in all comparisons.

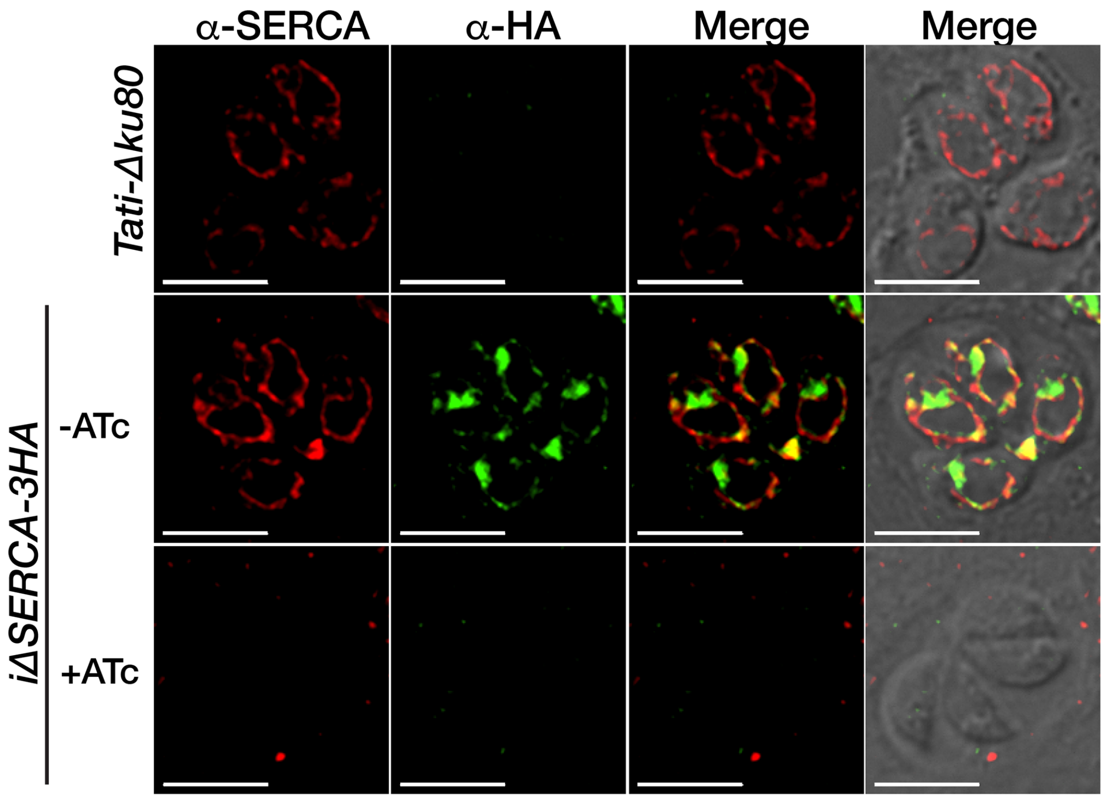

Figure S2: Regulation of the expression of TgSERCA. IFAs of *TatiΔku80* or *iΔTgserca-3HA* (±*ATc*). The mouse monoclonal antibody αHA was used at 1:25 dilution (green signal). The Guinea pig antibody against TgSERCA was used at 1:500 dilution (red signal). The HA signal co-localizes with the TgSERCA signal. They both disappear when the iΔTgSERCA-3HA cells were treated with ATc for 24 hours.

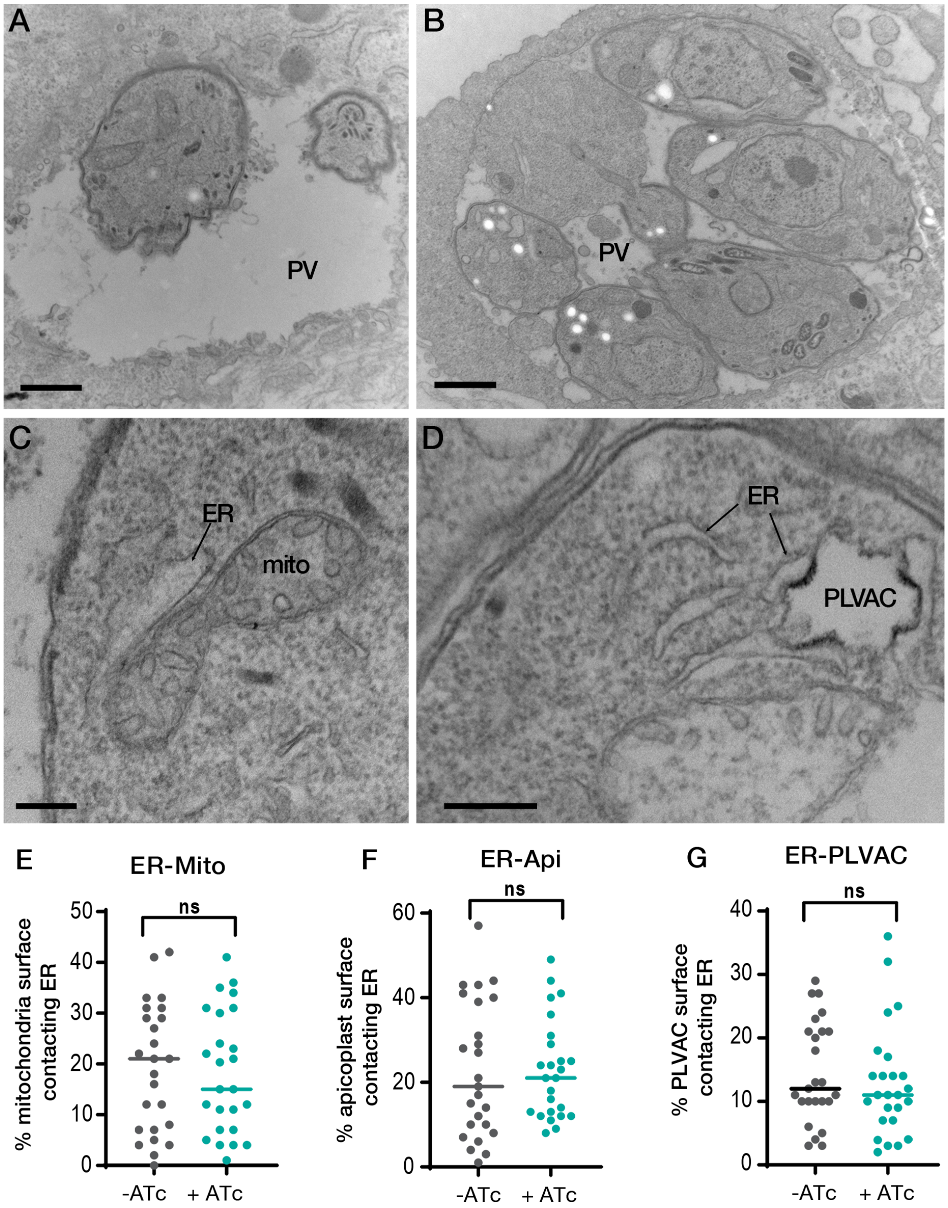

Figure S3: Electron microscope images of the *iΔSERCA* mutant treated with ATc for 24h. **A**, Representative images of the *iΔSERCA* mutant highlighting a large empty PV. Size bar is 500 nm. **B**, Representative image of the *iΔSERCA* mutant with a large residual body inside the PV. Size bar is 500 nm. **C**, The *iΔSERCA* mutant treated with ATc still showed contacts between the ER and mitochondria. Size bar is 100 nm. **D**, The *iΔSERCA* mutant treated with ATc still showed contacts between the ER and the PLV. Size bar is 100 nm. **E-G,** quantitative assessment of the contact area between ER and organelles (mitochondrion, apicoplast, or PLVAC), length of the limiting membrane of the organelle in contact with ER tubules at a distance less than 30 nm. This was measured and divided by the total length of the limiting membrane of the organelle. A total of 47 to 85 sections was analyzed for each population of organelles. E, Comparison of contacts measurements for *iΔTgSERCA* ± ATc for ER-mitochondria. F, Comparison of contacts measurements for *iΔTgSERCA* ± ATc for ER-apicoplast, C, Comparison of contacts measurements for *iΔTgSERCA* ± ATc for ER-PLVAC. All *p* values were calculated by two-tail t test comparing *iΔTgSERCA* ± ATc. *p* values ER-mito: 0.403; ER-Api: 0.492; ER-PLVAC: 0.244
